# Inferotemporal face patches are histo-architectonically distinct

**DOI:** 10.1016/j.celrep.2024.114732

**Published:** 2024-09-12

**Authors:** Hiroki Oishi, Vladimir K. Berezovskii, Margaret S. Livingstone, Kevin S. Weiner, Michael J. Arcaro

**Affiliations:** 1 Department of Psychology, University of California, Berkeley, Berkeley, CA, USA; 2 Department of Neurobiology, Harvard Medical School, Boston, MA, USA; 3 Department of Neuroscience, University of California, Berkeley, Berkeley, CA, USA; 4 Helen Wills Neuroscience Institute, University of California, Berkeley, Berkeley, CA, USA; 5 Department of Psychology, University of Pennsylvania, Philadelphia, PA, USA; 6 Senior author; 7 Lead contact

## Abstract

An interconnected group of cortical regions distributed across the primate inferotemporal cortex forms a network critical for face perception. Understanding the microarchitecture of this face network can refine mechanistic accounts of how individual areas function and interact to support visual perception. To address this, we acquire a unique dataset in macaque monkeys combining fMRI to localize face patches *in vivo* and then *ex vivo* histology to resolve their histo-architecture across cortical depths in the same individuals. Our findings reveal that face patches differ based on cytochrome oxidase (CO) and, to a lesser extent, myelin staining, with the middle lateral (ML) face patch exhibiting pronounced CO staining. Histo-architectonic differences are less pronounced when using probabilistic definitions of face patches, underscoring the importance of precision mapping integrating *in vivo* and *ex vivo* measurements in the same individuals. This study indicates that the macaque face patch network is composed of architectonically distinct components.

**In brief:**

Oishi et al. combine *in vivo* fMRI with *ex vivo* histology in the same macaque monkeys, revealing distinct histo-architectural differences among face patches, with the middle lateral (ML) face patch exhibiting the most pronounced differentiation, particularly in cytochrome oxidase staining.

## Graphical Abstract

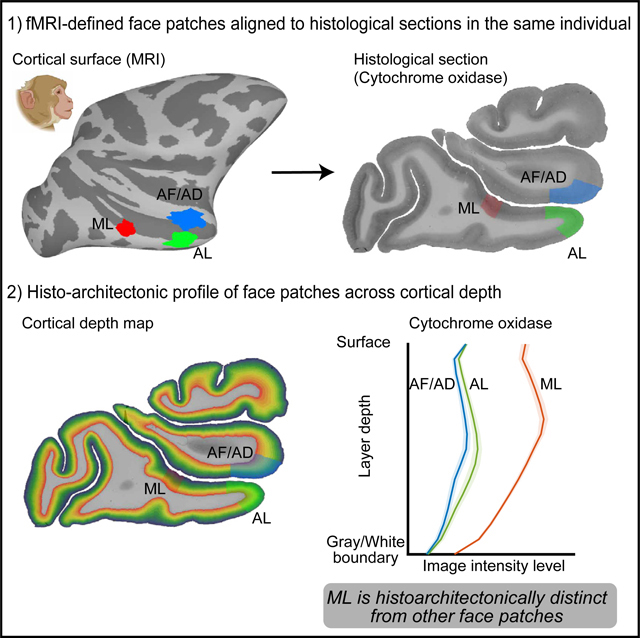

## INTRODUCTION

Understanding the microarchitecture of the cortex is essential for linking specific cellular and structural organization to observable behaviors and cognitive functions.^1,2^ Variations in cellular composition, density, and connectivity underpin the formation and functioning of cortical modules and networks, enabling diverse cognitive and sensory processes. Among primates, face recognition is a complex perceptual and cognitive process supported by a network of interconnected cortical regions, known as face patches, that are selectively responsive when viewing images of faces compared to images of other visual categories.^3–12^ While functional magnetic resonance imaging (fMRI) and electrophysiological studies have elucidated functional distinctions between face patches in the inferotemporal (IT) cortex,^13–15^ as well as how they develop,^16,17^ the histo-architectonic features that underlie these functional distinctions remain largely unexplored.

Resolving the histo-architecture of face patches can provide vital insights into the structural foundations of their specialized role(s) in visual perception. The current gap in knowledge persists because examining the ground truth of histo-architecture of functionally localized face patches in the same individual requires a unique combination of *in vivo* fMRI to localize face patches and postmortem histological measurements. Here, we solved this problem by developing a novel method to integrate fMRI and histological data from the same macaque monkeys. This approach enables us to quantify various histo-architectonic features of face patches (e.g., cytochrome oxidase [CO] and myelin) as well as their layer specificity (e.g., superficial vs. intermediate vs. deep layers).

These measurements uncovered a complex relationship between functionally defined face patches and the underlying histo-architecture, revealing three key findings. First, IT face patches differed from one another, and the surrounding IT cortex based on the intensity of their histo-architecture, with CO showing more pronounced differences than myelin. Second, CO staining was specifically stronger in the middle lateral (ML) face patch, a region thought to be homologous to the human fusiform face area(s),^6,9,10^ particularly in its outer layers. Third, the differences in CO staining were strongest when face patches were identified based on each animal’s fMRI activations rather than using a group probabilistic atlas of face patches, underscoring the precision of our methodological approach. These findings bridge the divide between macroscale functional neuro-imaging and microscale histological analyses in the same individuals.

Contextualized in the broader landscape of neuroanatomical research, our results draw parallels to classic studies that utilized myeloarchitecture and CO staining to inform histo-architecture and functional features of cortical areas, such as the parallel functional pathways of early visual processing.^18^ This illustrates the relevance of studying such anatomical architecture in the scope of high-level perceptual and cognitive processing. We anticipate that incorporating additional anatomical measures, such as local and long-range connectivity,^19^ into future research will deepen our understanding of the neuroanatomical foundations that underpin the functional diversity of the primate brain.

## RESULTS

### Deriving layer profiles of IT face patch histo-architecture

**Figure 1.**
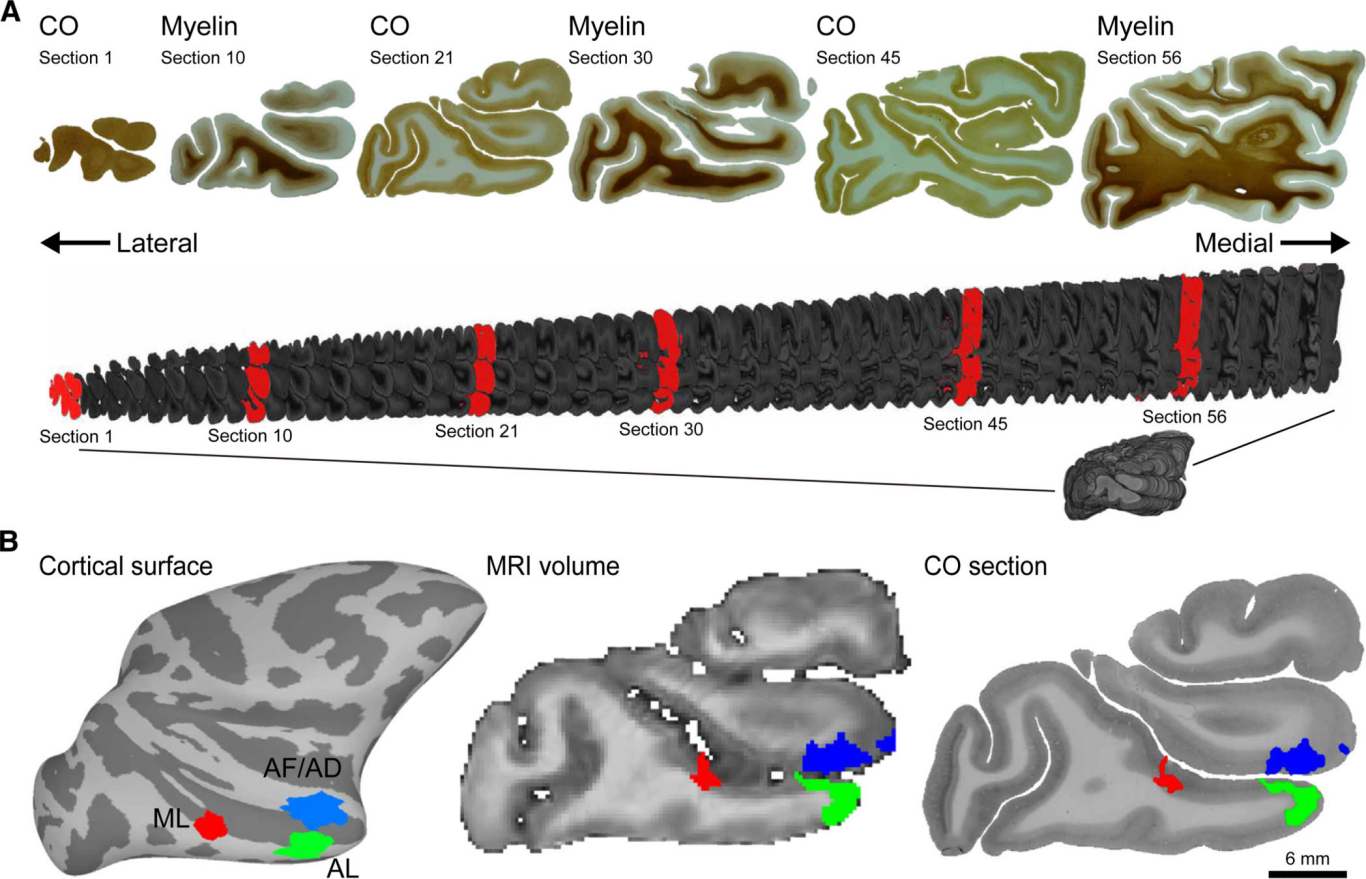
Projection of fMRI-defined face patches onto histological sections (A) Sequential sagittal histological sections from a right hemisphere in monkey M1. Sections were alternately stained for cytochrome oxidase (CO) and myelin. The histological sections were combined to construct a 3D histological volume (gray). The top representative sections correspond to the red sections in the 3D histological volume underneath (section 1, section 10, etc.). (B) To align and project fMRI-defined face patches onto histological sections, each monkey’s 3D anatomical MRI was coregistered to their 3D histological volume. Left: face patches on M1’s right hemisphere cortical surface. Face patches ML, AL, and AF/AD are shown in red, green, and blue, respectively. Center and right: face patches projected onto a single slice from M1’s anatomical MRI volume (center) and the corresponding histological section stained for CO (right). Videos scrolling in sagittal and coronal directions through the 3D histological volume and their aligned MR volume are provided (Videos S1 and S2).

To quantify the histological architecture of IT face patches, we acquired a unique dataset comprising fMRI, anatomical MRI, and histological data from the same individuals (*N* = 4 hemispheres from two monkeys). fMRI and anatomical MRI data were acquired *in vivo*. From the fMRI measurements, face-selective regions were identified as spatially contiguous regions along the cortical surface of the superior temporal sulcus (STS) that showed stronger activation when the monkeys viewed images of faces compared to other visual categories. Once these monkeys reached experimental endpoints, they were euthanized and perfused. After fixation, each hemisphere was cut into 50-μm-thick sections and alternately stained for CO and myelin (Figure 1A), which enables a comprehensive examination of both metabolic activity^20^ and axonal distribution.^21^ This comprehensive approach, combining functional and structural brain imaging with detailed histology, allowed for an in-depth analysis of the histological architecture of functionally defined face patches. While classic approaches have aimed to identify boundaries of cortical areas based on histo-architecture, our study focused on comparing histo-architectonic profiles of fMRI-defined faces patches.

To extract architectonic profiles of each face patch, we developed a novel pipeline to co-register 2D histological sections with 3D anatomical MR volumes and cortical surface reconstructions. The initial stage involved aligning adjacent brain sections using rigid body registration to preserve the global shape of the brain. In a second stage, histological sections were stacked and concatenated to reconstruct a 3D brain volume (Figure 1A). The final stage entailed aligning the anatomical T1 MRI volume with the reconstructed 3D histological volume (Figure 1B and Videos S1 and S2). The accuracy of this registration procedure was validated by calculating slice-by-slice image intensity correlations between the histology and MR volumes (Figure S1), where the mean correlations (r) between modalities were ≥ 0.835 for all hemispheres. Utilizing the high-resolution anatomical T1 MRI volume as a bridge, fMRI-defined face patches were mapped onto each histological section. This allowed for detailed assessment of the histo-architecture for each face patch (Figure 1B). Our analyses focused on three regions of face selectivity (ML, anterior lateral [AL], and anterior fundal [AF]/anterior dorsal [AD]) identified consistently in all histological samples.

**Figure 2.**
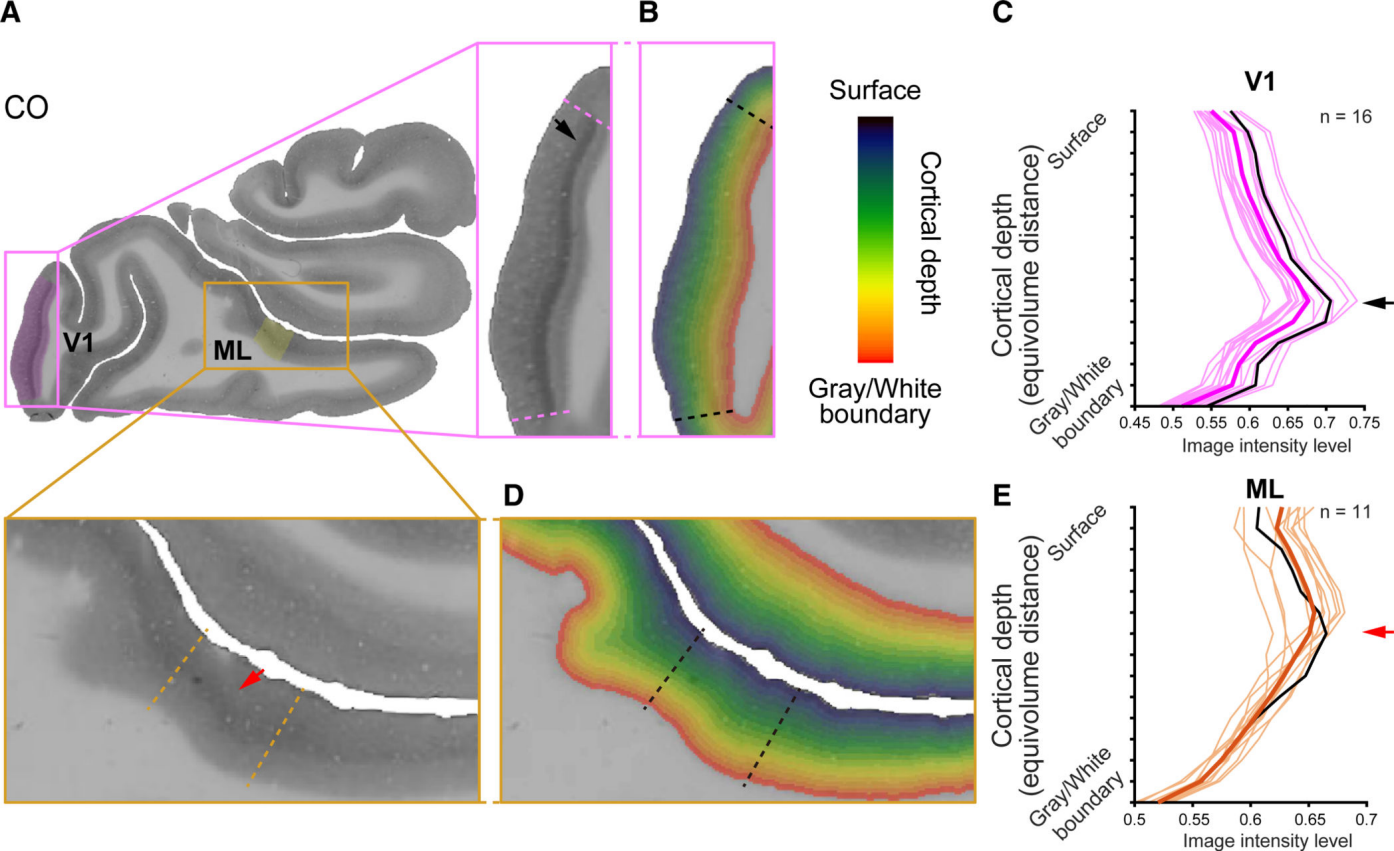
Architectonic profiles of fMRI-defined V1 and face patch ML from histological data in the same individual (A) Primary visual cortex (V1) and ML localized in the same histological section as shown in Figure 1B, with magnifications of V1 (pink boxes) and ML (orange boxes). Dashed colored lines illustrate the definitions of V1 and ML from fMRI data. (B) Cortical depth maps of V1 (pink) and surrounding cortex. (C) CO depth profiles across V1 sections. The pale pink curves illustrate CO profiles of each section, and the bolded pink curve illustrates the mean staining across sections. The black curve shows the depth profile for the section shown in (A). Higher numbers indicate darker staining intensity. The peak of the CO staining at mid-cortical depths (black arrow) corresponds to the visible thick band in V1 (A, black arrow). (D) Cortical depth map of ML (orange) and surrounding cortex. Conventions are consistent with (B). (E) CO staining across ML sections (*n* = 11). The pale orange curves illustrate the CO intensity of each section, and the bolded orange curve illustrates the mean intensity across sections. The black curve shows the depth profile for the section shown in (A). The dark staining toward mid-depths (red arrow) corresponds to the visible band of dark staining in ML (A, red arrow).

To analyze the histo-architecture of the face patches in relation to cortical depth, we segmented the cortical gray matter of each histological section into 15 equal-volume bins parallel to the cortex.^22–25^ The selection of 15 bins was determined by the architecture of layer IV in the primary visual cortex (V1), where this segmentation was the minimum required to consistently isolate the dark CO and myelin bands in layer IV^26,27^ to 1–2 bins across sections (Figures 2A–2C). This depth-based analysis allows for a detailed characterization of histological properties as a function of cortical depth but does not explicitly identify cytoarchitectonic laminae. Hereafter, we refer to the depth bin as “layers” for simplicity. The pixel intensities of CO and myelin images were averaged for each depth bin to construct histological layer profiles characterizing the variations in staining intensity across cortical depth for each face patch (Figures 2D and 2E).

Utilizing this pipeline, the depth architecture was assessed in the face patches and compared to V1. In both V1 and IT face patches, CO staining intensity increased from the superficial to middle layers, then decreased toward deep layers (Figures 2C and 2E). Notably, the peak (i.e., darkest) CO staining occurred in relatively superficial layers for the face patches as compared to V1 (Figures 2C and 2E), whereas the peak myelin staining intensity occurred at similar depths in V1 and the face patches (Figure S4). These areal differences were quantified by correlating the depth profiles across areas (mean correlation between face patches = 0.904 for CO and 0.957 for myelin; mean correlation between V1 and face patches = 0.508 for CO and 0.958 for myelin), revealing that the layer patterns of CO differ between face patches and V1, whereas those of myelin are similar across face patches and V1.

### ML is histologically distinct from other IT face patches

**Figure 3.**
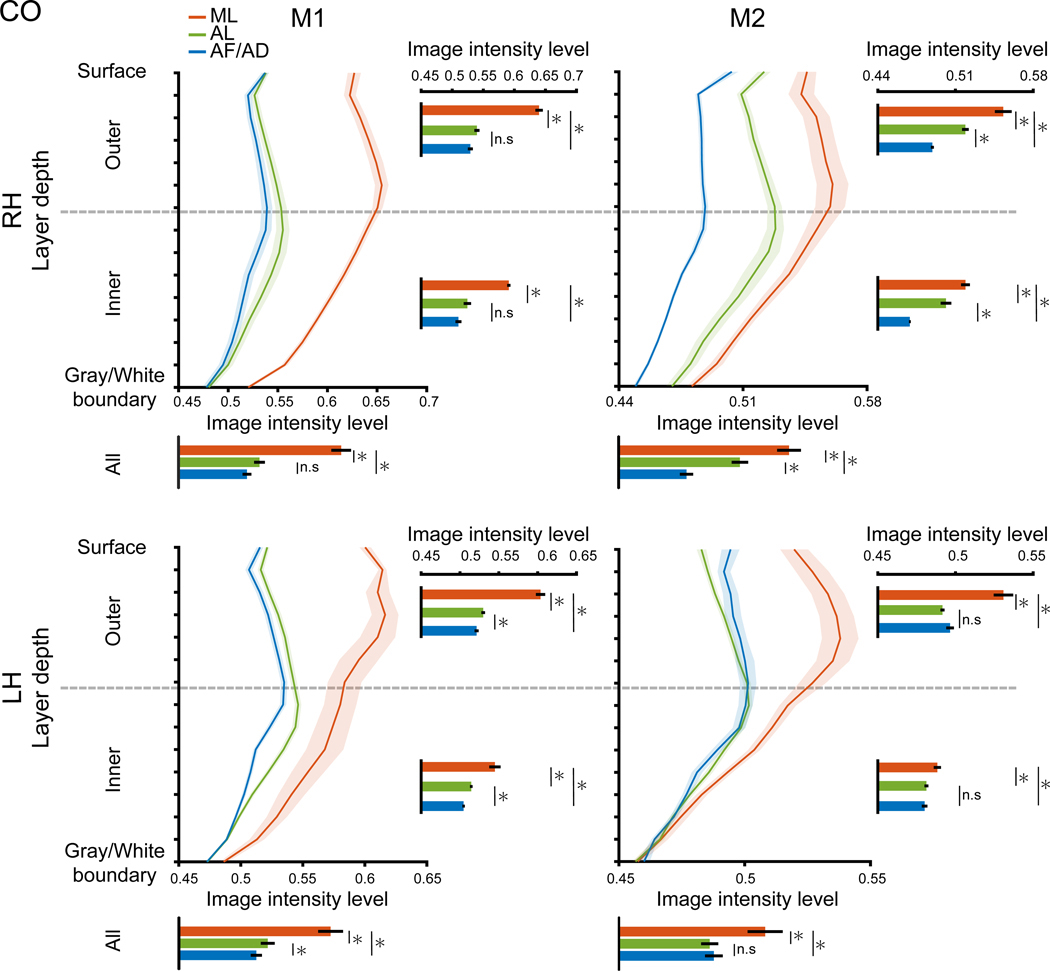
ML is architectonically distinct from other IT face patches CO stain intensities are plotted from the cortical surface, gray matter boundary (top) to the gray/white matter boundary (bottom). The curved lines represent the CO depth profiles of the three face patches measured in the present study (ML, red; AL, green; AF/AD, blue) in each hemisphere of M1 and M2. The shaded area represents ±1 SEM across histological sections. Higher numbers indicate darker staining intensity. The horizontal bar graphs to the right of each depth profile show the mean stain intensities across outer layers (top seven depth bins) and inner layers (bottom eight depth bins). The mean stain intensities across all layers are shown below each depth profile (error bars, ±1 SEM). Asterisks indicate significant differences between face patches (*p* < 0.05, false discovery rate [FDR] corrected). The statistics of all comparisons are shown in Table S1.

While the pattern of staining across layers was similar among face patches, the degree of staining intensity varied among them. To assess this, we first calculated the mean depth profile averaged across histological sections for each face patch (see Figure 3 for CO architecture and Figure S4 for myeloarchitecture). These depth profiles characterize the variability in staining across layers for each face patch. Then, we tested whether the depth profiles across face patches differed in three ways: (1) collapsed across layers, (2) in outer layers, and (3) in inner layers. To collapse across layers, staining intensities were averaged across depth bins per section, which were then compared across face patches using unpaired two-sample t tests. The comparisons revealed that CO intensities in ML were significantly higher than those in the AL and AF/AD for all hemispheres (all ts > 4.223, all ps < 0.05 [false discovery rate (FDR) corrected for multiple comparisons] in ML compared to AL and ML compared to AF/AD; Figure 3; see Table S1 for the statistics of all face patch comparisons). Significant differences were observed for myelin in all but four comparisons, though the numerical differences intensities were smaller (Figure S4; Table S1). AL and AF/AD face patches were differentiated less clearly, being significant only for 2 of 4 and 3 of 4 hemispheres for CO and myelin staining, respectively (ts > 2.437 for the significant comparisons [ps < 0.05]; Figures 3, S4, and S6; Table S1) and varied across hemispheres in terms of which area had darker staining. Given this variability, comparisons between AL and AF/AD were not considered for subsequent analyses. For the comparisons in outer and inner layers, staining intensities were averaged across the upper seven and lower eight depth bins, respectively. Consistent with the analyses collapsed across layers, differences across face patches extended to both outer and inner layers for CO (all ts > 2.559, all ps < 0.05; Figure 3; Table S1), but this was not observed consistently for myelin (Figure S4; Table S1). These results revealed differences in the depth architecture between face patches, particularly for CO.

**Figure 4.**
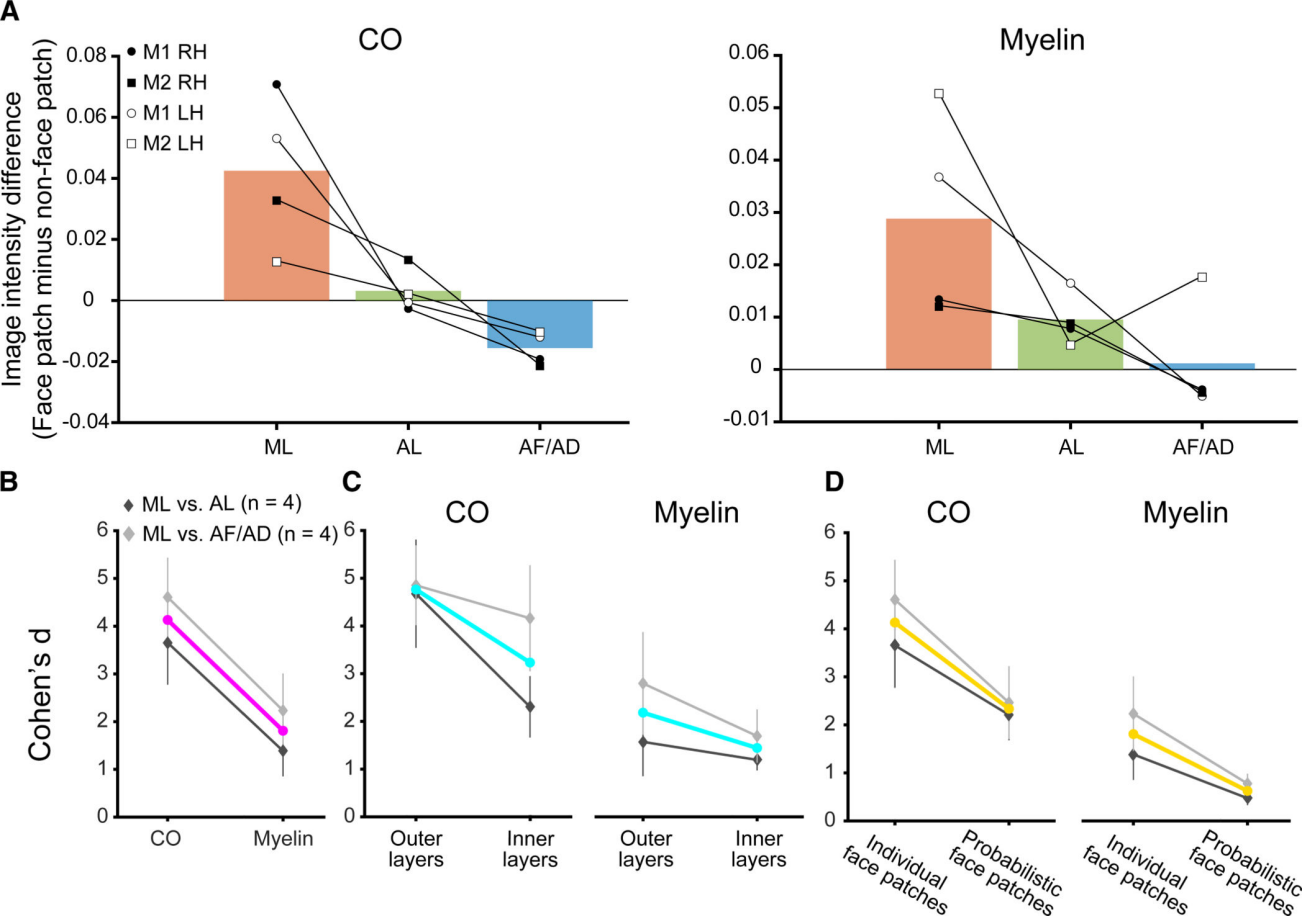
Architectonic differences between ML and other face patches are strongest for CO in outer layers in individual participants (A) Image intensity differences between face patches and neighboring STS regions outside of face patches (non-face patch). Data points (*N* = 4 hemispheres) shown as filled and empty circles and squares, with bars representing the mean across hemispheres. Intensity differences are significant for ML vs. AL and ML vs. AF/AD in all hemispheres (ts > 2.692, ps < 0.001 for CO and myelin, using unpaired, two-sample t tests [FDR corrected for multiple comparisons]). See Figure S2 for individual hemispheric data. (B) Mean effect sizes (Cohen’s d) for CO and myelin across all hemispheres/monkeys (error bars, ±1 SEM) for ML vs. all other face patches (magenta), ML vs. AL (*n* = 4, dark gray), and ML vs. AF/AD (*n* = 4, light gray). Cohen’s d for CO was significantly higher compared to myelin in 7 of 8 pairs for each comparison (Figure S5A). (C) Mean Cohen’s d for outer and inner depths across all hemispheres/monkeys (error bars, ±1 SEM) for CO (left) and myelin (right) for ML vs. all other face patches (cyan). Cohen’s d was significantly higher in the outer layers compared to the inner layers for CO in 7 of 8 pairs and 5 of 8 pairs for myelin (Figure S5B). The depth bins corresponding to the outer and inner depths are shown in Figure 3. The other conventions are consistent with (B). (D) Cohen’s d was significantly higher for individually defined compared to probabilistically defined face patches in CO and myelin (both significant for 7 of 8 pairs; Figure S5C). Each line represents Cohen’s d collapsed across hemispheres and monkeys for individually and probabilistically defined face patches, shown in gold. The other conventions are consistent with (C). All statistics for each pairwise comparison for (B)–(D) are shown in Tables S2, S3, and S4.

As histo-architecture tends to vary gradually across the macaque cortex,^28^ we tested whether the histological differences between face patches are due to the broader histological variability along the STS. We generated columnar bins orthogonal to the cortical surface along the STS and calculated the mean staining intensity within each columnar bin, collapsing across layers (see STAR Methods for the columnar bin generation). Variations in histological intensities were assessed along a 2D plane (dorsolateral-ventrolateral axis × posterior-anterior axis, Figure S2; see Figure S3 for the 1D plots collapsed for the two axes). While histological intensities varied across both axes, histological differences between face patches remained significant even after subtracting the histological intensities of face patches by the intensities outside of face patches at corresponding sagittal and coronal locations within IT (ts > 2.692, ps < 0.001 for CO and myelin in all hemispheres; Figure 4A). This indicates that the CO and myelin variability between face patches is not the result of broad variations in the staining intensity along the posterior vs. anterior or dorsomedial vs. ventrolateral axes.

To determine which staining method best differentiated the histological architecture among face patches, we compared the effect size (Cohen’s d) between CO and myelin. To make statistical comparisons despite having a limited number of animals, we calculated bootstrap-generated Cohen’s d for each pair of face patches (STAR Methods). This approach allowed differences in the distributions of Cohen’s d between CO and myelin staining for the same pairs of face patches to be tested using a Kolmogorov-Smirnov (KS) test. The ML and the other face patch depth profiles were more discriminative in CO compared to myelin (Cohen’s d Δ[CO vs. myelin] > 1.137, KS distances > 0.799, ps < 0.001 for seven of eight comparisons; Figures 4B and S5A; Table S2).

Additionally, we tested whether the discriminability between ML and the other face patches varied with cortical depth. To do so, we compared the bootstrap-generated Cohen’s d between staining intensities within outer and inner layers. The effect sizes for ML compared to the other face patches were significantly stronger in outer compared to inner layers for CO (Cohen’s d Δ[outer vs. inner layers] > 0.527, KS distance > 0.366, ps < 0.001 for seven of eight pairs; Figures 4C and S5B; Table S3). For myelin, the effect sizes for ML compared to the other face patches were only significantly stronger in outer compared to inner layers for a subset of comparisons (Cohen’s Δ > 0.522, KS distance > 0.271, ps < 0.001 for 5 of 8 comparisons; Figures 4C and S5B; Table S3). These results illustrate that face patches were more distinct based on staining in their superficial layers, particularly for CO.

Finally, to assess group effects and the impact of variability across monkeys and hemispheres, we compared the mean depth profiles of the four hemispheres between face patches (*n* = 60 [15 × 4, depth bins × hemispheres] for each face patch). The normal distribution curves fitted to the face patches are depicted in Figure S6. Across monkeys and hemispheres, ML was distinct from the other face patches for CO but not myelin (KS distance [*p* value] for ML vs. AL and ML vs. AF/AD distributions = 0.467 [*p* < 0.001] and 0.617 [*p* < 0.001] for CO, 0.200 [*p* = 0.192] and 0.233 [*p* = 0.097] for myelin, using KS test). Together, these results show that the CO architecture of ML is reliably distinct from AL and AF/AD across our sample.

### Larger effect sizes when identifying face patches based on individual data as compared to probabilistic definitions

The present study leveraged unique data that included fMRI-localized face patches and postmortem histology from the same animals. When brain regions cannot be defined in individual animals, an alternative option is to use a probabilistic atlas derived using functional and/or anatomical data from a separate group of individuals. However, such atlases are inherently limited by individual variability in structure and function correspondences.^29,30^ The impact of this variability on the accuracy of using probabilistic definitions to probe histological architecture has yet to be examined.

To assess the potential advantage of our approach over traditional atlases, we generated probabilistic definitions of face patches using data from an independent group of monkeys (*N* = 5).^30^ Probabilistic face patches were defined on the National Institute of Mental Health macaque template and then projected onto the histological volumes of the individual hemispheres from each monkey, leading to two key findings. First, ML was still found to be architectonically distinct from AL and AF/AD when using the probabilistic definition (Figure S7), consistent with prior observations that face patches are typically found in similar anatomical locations across monkeys.^29,30^ Second, the effect sizes were stronger when using individually defined face patches in both CO and myelin (Cohen’s d Δ[individually vs. probabilistically defined face patches] > 0.192, KS distance > 0.226, ps < 0.001 for seven of eight pairs for CO; Cohen’s d Δ > 0.028, KS distance > 0.029, ps < 0.001 for all pairs for myelin, using KS test; Figures 4D and S5C; Table S4). The diminished effect when using probabilistic definitions likely reflects individual variability in the anatomical localization of face patches on the order of several millimeters.^30^ This variability was reflected in a modest overlap between individual and probabilistic face patches (mean Dice coefficient [collapsed across face patches] = 0.578 for right hemisphere of M1, 0.493 for right hemisphere of M2, 0.217 for left hemisphere of M1, 0.430 for left hemisphere of M2). As a result, the histo-architecture of face patches was less distinct with probabilistically defined face patches. Together, these results validate the use of probabilistic definitions for investigating the histological architecture of face patches but empirically show that (1) the effect size will be underestimated compared to using individually defined data and, (2) depending on the specificity of the architecture being compared, may be missed entirely.

## DISCUSSION

Our results revealed architectonic distinctions among IT face patches. The most notable distinction was the quantitative differentiation of ML from other face patches in the IT cortex, primarily based on CO, particularly in the outer layers, and, to a lesser extent, based on myelin. Here, we discuss these findings with previously identified anatomical and functional features that set ML apart from other face patches, which enhances our understanding of ML’s anatomical and functional specialization as well as its potential homologies with human face patches.

### On the microarchitecture of ML: Classic histology of IT, probabilistic areal definitions, and “precision imaging”

While our study is the first to identify architectonic differences using histological staining techniques on fMRI-defined face patches, the temporal lobe has been historically parcellated based on various architectonic features, finding that ML is typically located within the broadly defined posterior TE that extends along the lower lip of the STS.^6^ However, there are disagreements in the precise subdivisions of temporal areas where ML is positioned due to the variability of areal definition methods. For example, Janssens and colleagues compared the probabilistic location of ML to probabilistic definitions of cortical areas based on connectivity^31,32^ or multiple architectonic features (myelin, Nissl, AChE, SMI-32, and CAT-301^33,34^). These comparisons showed that, while ML is largely anatomically distinct from other areas, it falls amidst what Charles Gross described as the “alphabet soup” of cortical areas, owing to differences in parcellations across research groups.^35^ That is, according to these probabilistic definitions, ML is located in portions of (1) PITd and CITd,^32^ (2) FST, TEm, and TE3,^34^ (3) FST and TE,^31^ and/or (4) TEa/m and IPa.^33^ Consistent with this latter probabilistic finding, Baylis et al. showed that about 20% of neurons were face selective (measured premortem) in histologically defined (postmortem) TEa and TEm in the same animals.^36^ Lewis and Van Essen noted that contributing to the variability in areal distinctions across multiple parcellations is that some methods are more sensitive to revealing the distinct architectural features within this portion of IT than others. For example, Lewis and Van Essen write: “Architectonic subdivisions ventral to the STS and along the ventral temporal lobe were based exclusively on cyto- and myeloarchitecture (see Table 2), as SMI-32 and CAT-301 immunoreactivity was too sparse to reliably differentiate boundaries’ (pg. 100).”^33^ Therefore, the present work overcomes this difficulty, as well as complements prior work, by first identifying functional face patches in the IT cortex and then examining the histo-architecture of each face patch.

Our approach aligns with the concept of “precision” or “deep” imaging in the human neuroimaging field that emphasizes the importance of using individual, subject-specific measurements compared to probabilistic atlases.^37–41^ Our results show that architectonic differences between face patches are underestimated when using probabilistic regions of interest compared to individually defined face patches (Figures 4D and S5C; Table S4). An alternative option to the present approach is measuring fMRI and structural MRI that are related to histo-architecture in the same animal. For example, quantitative MRI and T1/T2 metrics are related to myelin content,^42–48^ with some discrepancy as to which MR metrics most accurately match histology.^49^ However, to our knowledge, no MR sequences can identify other architectonic features, including CO architecture, unless those features are correlated with myelin content. Considering that ML most clearly differed from other face patches in CO staining and less in myelin, it is likely that such MR-based architectonic mapping would not have revealed architectonic differences.

### Relating the CO profile of ML to the functional specialization of ML

To our knowledge, few studies have examined the CO organization of the temporal lobe. One previous study focused on areas in the dorsal portion of the STS, such as middle temporal visual area, FST, MST, and MTc in galagos,^50^ but did not specifically investigate the CO organization of the IT cortex and the anatomical regions where face patches are typically localized. As CO is a functional architectonic measure, here, we relate the CO profile to known functional properties of ML.

Functionally, ML is located just anterior to the retinotopically defined area PITd in the foveally biased cortex.^29,51^ As CO has been related to fovea-related processing in early visual areas,^52^ the difference in the CO profile of ML compared to other face patches measured here could be related to differences in receptive field size and a bias for foveal processing. It could also be related to differences in face processing. For instance, inactivation of ML indicates that it is causally involved in face detection^53^ and identification.^54^ Additionally, ML neurons are view specific,^14^ further distinguishing it functionally from more anterior face patches. Previous studies had difficulty differentiating ML from the anatomically nearby face patch, MF, which was excluded in the present study due to lack of coverage in the sagittal sections (2/4 hemispheres). Thus, it remains possible that MF exhibits either a similar or different CO profile than ML, which can be explored in future studies with both areas mapped in the same animals.

### The CO architecture of ML and local microcircuitry

To our knowledge, the only prior study to examine postmortem anatomical features of face patches that were first localized with fMRI was conducted by Grimaldi et al., focusing on the connectivity patterns of each face patch.^19^ Based on earlier work linking feedforward and feedback connections to specific layers of the cortex^1^ and layer-specific connectivity acting as a proxy for an area’s position in a cortical hierarchy,^32,55,56^ their study concluded that none of the face patches exhibited a clear feed-forward connectivity scheme. This is in apparent contrast to ML’s physical location anterior to the extrastriate area V4 and posterior to AL, AF, and AD, which might indicate an intermediate position for ML in the ventral visual hierarchy. Particularly relevant for the current study, ML was connected with a large number of cortical regions, including AM, AF, PL, contralateral ML, V4v, orbitofrontal area (Brodmann area 13 m/l), as well as scattered clusters of cells between AF and MF. Subcortically, ML is also connected to the claustrum and pulvinar in clusters that are non-overlapping relative to other face patches. Such extensive connectivity with other visual regions could be reflected in enhanced staining for CO, serving as an endogenous marker for neuronal activity.^27^ Thus, while ML does not have a clear feedforward/feedback connectivity scheme with other face patches or cortical regions outside the face network, the CO organization of ML could also relate to differences in local microcircuitry, which can be explored in future studies.

### Limitations of the study

While the present study focused on CO and myelin stains, other stains (e.g., Nissl, SMI-32, and calbindin) could further elucidate histo-architectonic differences among face patches, potentially revealing additional insights into laminar architecture and subcortical connectivity. Further, while our sample size is comparable to other nonhuman primate (NHP) studies, future studies with larger sample sizes (when available, given the rarity of these data) could test additional novel questions, such as potential laterality differences in the face patch histo-architecture, given the present controversy regarding the laterality of face processing in NHPs.^12^

Advanced machine learning algorithms could improve the alignment of the MRI and histology data.^57^ Furthermore, while the present study focused on comparing the histo-architecture of face patches in NHPs, extending this pipeline to other category-selective networks could provide valuable insights into the broader histo-architectonic similarities and differences of cortical networks selective for processing different categories.^58–60^ Finally, a future, albeit ambitious,- study comparing the histo-architectonic features of face patches between humans and NHPs, with fMRI premortem and histology postmortem in the same individuals, will further shed light on the evolution of face processing network microarchitecture.

Altogether, this study represents the first quantitative analysis of histo-architecture across cortical depths in fMRI-localized macaque face patches. By combining *in vivo* fMRI with *ex vivo* histology in the same macaque monkeys, we revealed that face patches differed in their histo-architecture, with the largest difference between ML (considered a potential homolog to the fusiform face area[s]) and the other face patches, especially for CO, and less so for myelin. As ML is functionally distinct from, and differs in connectivity compared to, other face patches, the histo-architectonic difference compared to other face patches identified in the present study likely contributes to these functional and connectivity differences of ML compared to other face patches. Our findings underscore the importance of integrating multiple levels of analysis to understand the neural substrates of functional networks, providing a foundation for future investigations of the relationship between cortical microstructure and functional specialization in the primate visual system.

## RESOURCE AVAILABILITY

### Lead contact

Further information and requests for resources should be directed to and will be fulfilled by the lead contact, Hiroki Oishi (hoishi@berkeley.edu).

### Materials availability

This study did not generate new unique reagents.

### Data and code availability

All histological sections and the aligned face patches are publicly available in the repository (key resources table). This repository also contains the code for generating the histo-architectonic depth profiles of the face patches. Any additional information required to reanalyze the data reported in this paper is available from the lead contact upon request.

## STAR★METHODS

### EXPERIMENTAL MODEL AND STUDY PARTICIPANT DETAILS

#### Monkeys

All training, surgery, and experimental procedures were approved by the Harvard Medical School Animal Care and Use Committee and conformed with NIH guidelines for the humane care and use of laboratory animals. Two adult monkeys (Macaca mulatta) underwent T1w and functional MRI and histological experiments. Six adult monkeys underwent T1w and functional MRI, whose data were used for creating the probabilistic face patches.

### METHOD DETAILS

#### Anatomical MRI

As previously reported,^51^ a whole-brain structural volumes were acquired using a 3 T Siemens Skyra scanner equipped with a 15-channel transmit/receive knee coil while the animals were anesthetized with a combination of Ketamine (4 mg/kg) and Dexdomitor (0.02 mg/kg). Monkeys were scanned using a magnetization-prepared rapid gradient echo (MPRAGE) sequence; 0.5× 0.5 × 0.5 resolution; field of view = 128 mm; 256 × 256 matrix; repetition time (TR) = 2,700 ms; echo time (TE) = 3.35 ms; inversion time (TI) = 859 ms; flip angle = 9°. Whole-brain T1-weighted anatomical images were collected from each animal.

##### Gray matter segmentation and cortical surface reconstruction

T1w anatomical MRI images were used to co-register with functional anatomical MRI (fMRI) data (*in vivo*) and histological data (postmortem), as well as to define regions of interest (ROIs) on cortical gray matter. To do so, each animal’s T1w MRI images were co-registered to derive an average anatomical volume image for each monkey (seven scans for M1, two scans for M2). Each monkey’s average anatomical volume underwent semi-automated cortical surface reconstruction using FreeSurfer. To ensure high accuracy, skull stripping and white matter masks were first manually segmented by an expert slice-by-slice along coronal, axial, and sagittal planes and then passed into FreeSurfer’s autorecon pipeline. Pial and white-matter surfaces were inspected to ensure accurate segmentation. If poor segmentations were detected, the white-matter mask and control points were edited, and surface reconstruction was rerun until corrected. For several monkeys, FreeSurfer’s auto-segmentation had trouble with the calcarine and highly vascularized regions such as the insula. To fix these segmentation errors, average anatomical volumes were manually edited to improve the gray/white-matter contrasts and remove surrounding non-brain structures (e.g., sinuses, arachnoid, and dura-mater).

#### Functional MRI

As previously reported,^51^ monkeys were scanned in a 3-T Tim Trio scanner with an AC88 gradient insert using four-channel surface coils (custom made by Azma Maryam at the Martinos Imaging Center, Charlestown, MA). Each scan session consisted of 10 or more functional scans. We used a TR of 2 s, TE of 13 ms, flip angle of 72°, integrated parallel acquisition techniques = 2, 1 mm isotropic voxels, matrix size 96 × 96 mm, and 67 contiguous sagittal slices. To enhance contrast,^61,63^ we injected 12 mg/kg monocrystalline iron oxide nanoparticles (MION; Feraheme, AMAG Pharmaceuticals) in the saphenous vein just before scanning.

##### Stimuli

Responses to image categories of faces and inanimate objects were probed as previously reported.^51^ Each scan was composed of blocks of each image category; each image subtended 20° × 20° of the visual angle and was presented for 0.5 s; block length was 20 s, with 20 s of a neutral gray screen between image blocks. Blocks and images were presented in a counterbalanced order. All images were centered on a pink noise background. All images were equated for spatial frequency and luminance using the SHINE toolbox.^63^

##### Data preprocessing

Functional scan data were analyzed using AFNI,^61^ SUMA,^62^ Freesurfer,^64,65^ JIP Analysis Toolkit (https://www.nitrc.org/projects/jip), and MATLAB (Mathworks). Each scan session for each monkey was analyzed separately. Using AFNI, all images from each scan session were aligned to a single time point for that session, detrended, and motion corrected. Data were spatially filtered using a Gaussian filter of 2 mm full width at half-maximum to increase the signal-to-noise ratio while preserving spatial specificity. Each scan was normalized to its mean. Data were registered using a two-step linear and then a nonlinear alignment approach (JIP Analysis Toolkit) to a high-resolution (0.5 mm) anatomical image for each monkey. First, a 12-parameter linear registration was performed between the mean EPI image for a given session and a high-resolution anatomical image. Next, a nonlinear, diffeomorphic registration was conducted. To improve registration accuracy of the ventral cortex, we manually drew masks that excluded the cerebellum for both EPI and anatomical volumes prior to registration.

##### Individual monkey face-patch localization

A multiple regression analysis (AFNI’s 3dDeconvolve) in the framework of a general linear model was performed on the responses to face and inanimate object image categories for each monkey separately. Each stimulus condition was modeled with a MION-based hemodynamic response function.^66^ Additional regressors that accounted for variance due to baseline shifts between time series, linear drifts, and head-motion-parameter estimates were also included in the regression model. Face-selectivity was defined as regions that responded more strongly to images of faces compared to inanimate objects. Maps of beta coefficients were clustered (>10 adjacent voxels), and the threshold was at *p* < 0.0001 (false discovery rate [FDR]-corrected). In accordance with previous work,^30^ the middle lateral (ML), anterior lateral (AL) and anterior fundal (AF) and anterior dorsal (AD) face-selective patches within the superior temporal sulcus (STS) were defined. Activity was spatially continuous between AF and AD and these regions were grouped for all analyses. While additional face patches have been reported, we limited the analyses to the three face-patch regions (ML, AL and AF/AD) which were consistently identified in the histological sections of all hemispheres.

##### Probabilistic face-patch mapping

As previously reported,^30^ probabilistic maps of ML, AL, and AF/AD face patches were created by averaging the face-patch ROIs from an independent group of monkeys (*N* = 5) in the National Institute of Mental Health macaque template (NMT) brain space.

#### Histology

As previously reported,^30^ monkeys that naturally reached endpoints were euthanized by intravenous injection of SomnaSol (dose of sodium pentobarbital was 120 mg/kg), and transcardially perfused by rinse (0.9% sodium chloride +0.5% sodium nitrite) followed by 4% paraformaldehyde in 0.1 M phosphate buffer, pH 7.4. After postfixation overnight, the brain was placed into 30% sucrose in 0.1 M phenobarbital. The processed brains were sectioned aiming to cover the STS. The left hemispheres were sectioned in the coronal plane while the right hemispheres were sectioned in the sagittal plane. Both sections were cut at 50-mm thickness in freezing microtome. Serial sections, mounted on glass slides and postfixed for 12 d in formol saline (10% formalin +9 g/L sodium chloride), were processed for cytochrome oxidase (CO) according to a standard technique (key reagents: Catalase, Cytochrome *c* and 3,3´-diaminobenzidine).^67^ Another batch of free-floating sections was stained for myelin by the Gallyas method (key reagents: Silver nitrate, Ammonium nitrate, Sodium hydroxide, Sodium carbonate, Sodium thiosulfate and Tungstosilicic acid hydrate).^21^ The slices were stained for CO and myelin alternatively slice-by-slice. While a few slices were stained for a different staining method, we excluded them for the histo-architectonic analyses due to the small number of the slices. Digital images of stained sections were captured using a Panasonic Lumix DMC-ZS7 camera with a 12X optical zoom and a light box for uniform lighting.

##### Section co-registration and creation of 3D histology volume

Using Illustrator software (Adobe System Inc.), non-brain pixels were set to 0 to segment the brain and the images were converted to 8-bit gray scales. Images were downsampled to 768 × 1024 pixels for sagittal sections and 1024 × 768 pixels for coronal sections, which minimized memory processing requirements while maintaining a sufficient resolution to measure variability in stain intensity across cortical depth.

Co-registration of histology slices proceeded in two stages using the Image Processing Toolbox in MATLAB. First, sections were co-registered based on their global shape. To achieve this, sections were converted into binary images where pixels within the brain were assigned a value 1 and pixels outside were assigned a value 0. For each hemisphere, a middle section was chosen as the target reference image. Adjacent sections were co-registered in a serial fashion ascending and descending from the reference such that after the adjacent sections were co-registered to the reference, the resulting transformed sections served as the reference for the next set of adjacent sections. Sections were co-registered using a 6-parameter rigid body transformation. In the second stage of the alignment, the cortical gray was segmented from the white matter and subcortex in each section by applying a multilevel thresholding method^68^ to the histograms of image intensities. Gray and white matter pixels were set to values of 127 and 255, respectively. The resulting segmented sections were then co-registered using a multimodal, rigid body transformation. The gray matter masks were also used in the cortical depth analysis. The transformations from both stages were applied to the original CO and myelin sections. Sections were stacked to create a 3D volume of CO and myelin. The section axis was interpolated to preserve the spacing between sections.

To mitigate artifactual image intensity variability across histological sections that could arise during the staining process, we calculated the mean intensity for each section, fit a quadratic function to the mean intensities across all sections, and then adjusted the intensities of each section based on the deviation of their mean intensity from the fit. This correction allowed for gradual drifts in the mean intensity across slices that will arise from varying ratios of gray and white matter across sections while enforcing neighboring slices to have similar mean intensities. This intensity correction was performed to the CO and myelin sections separately.

##### Alignment between anatomical MRI and histological data

To project the fMRI-defined face patches to histological data, we co-registered each 3D histological volume to the corresponding monkey’s T1 anatomical MRI volume. We first resampled histological volume to match the MRI resolution. We then trimmed T1w MRI data to match the content of the histological volume. After that, we aligned both data using the JIP toolkit, which performs first affine alignment and then non-linear alignment. To evaluate the alignment accuracy, we calculated the correlation of image intensities between the aligned T1 MRI and histological data slice by slice (Figure S1). We compared these correlations to the correlations between non-corresponding slice pairs. The correlation between the aligned multiple T1 data of the monkey M1 were calculated to check the practically highest correlation between brain images (Dashed horizontal line in Figure S1).

##### Projections face patches in MRI space to histological data

We warped the face patches on MRI data onto the resampled histological data using the transformation matrix between MRI and resampled histological data. After that, the face patches were upsampled to the resolution of the original histological data. Since the warps change the shape of a patch, some parts within the projected patch did not fully cover the whole cortical depths. Thus, when localizing the face patch on the histological sections, we extended these parts perpendicularly to cover the whole cortical depths. The probabilistic face patches were projected by aligning the NMT brain to the individual histological volume, and then localized in the same way as the individual face patches.

##### Generating iso-layers across cortical depths

To investigate the microarchitecture across cortical depths, we segmented the cortical gray matter using the LN2_LAYERS function from LayNii (https://github.com/layerfMRI/LAYNII).^22^ In preparation for performing LayNii, we generated gray matter masks of the outer surface and the gray and white matter boundary in each histological section. The gray matter masks were created using the MATLAB implementation of Otsu’s multilevel image thresholding,^68^ then we defined the surface and bottom boundaries of gray matter automatically by identifying gray matter pixels which are adjacent to the background (i.e., out of the brain) and white matter, respectively. The gray matter was segmented in accordance with the equi-volume principle,^23^ which takes into account that outer layers have thinner volume^22–25^ and produces depth bins with the same volume. The cortical gray matter was segmented into fifteen equi-volume bins (colored bins in Figures 2B and 2D). This number of the depth bins was sufficient for isolating the prominent staining intensities in layer IV in V1 (Figures 2A–2C) to 1–2 bins across sections.

### QUANTIFICATION AND STATISTICAL ANALYSIS

#### Anatomical histo-architectonic analysis

##### Histological depth profiles

For each myelin and CO histological section, pixel values were normalized and rescaled from a range of 0–255 to 0–1, where 0 corresponds to no staining and 1 corresponds to the darkest staining. For each histological section, staining intensities were averaged within each depth bin to generate the depth profiles for each ROI (i.e., V1 and face patches; Figures 2C and 2E). The mean depth profile and variance across histological sections (bold pink and orange lines in Figures 2C and 2E) was computed for each ROI.

##### Laminar pattern similarity analysis

The laminar patterns of histological stains were compared across our regions of interest by correlating the mean laminar profiles for each ROI. For V1, this analysis was restricted to the right hemisphere sagittal sections (*n* = 2) as coronal sections only covered parts of the STS. Correlations were conducted between monkeys for corresponding ROIs as well as between ROIs in the same monkey.

##### Statistical comparisons of histology

Each face patch profile was averaged across depth bins to obtain the single intensity measure for each histological section (section intensity). The section intensities were compared across face patches using unpaired, two-sample t-tests (Figures 3, S4, and S7). To test for differences across cortical depth, this comparison was repeated for mean intensities derived from the outer and inner depth segments separately. Outer and inner layer intensities were calculated by averaging the upper seven and lower eight depth bins of the cortical depth-based profiles. FDR correction was applied to account for multiple comparisons.

##### Group-level comparisons

Probability density functions (PDF) were fitted on the mean depth profiles across all sections and animals for each face patch to compare the means and standard deviations between ROIs at the group level (Figure S6). The group level distributions of face patches were visualized by using the mean depth profiles of all hemispheres. Their differences were tested by the KS test between the mean depth profiles of all hemispheres among face patches (FWD-corrected).

##### Bootstrap analysis to compare discriminability across contrasts

We tested the discriminability of the staining intensity for all face-patch pairs (ML vs. AL, ML vs. AF/AD, AL vs. AF/AD, ns = 4 for each) for three contrasts: (1) CO vs. myelin (Figures 4B and S5A; Table S2), (2) outer vs. inner layers (Figures 4C and S5B; Table S3), and (3) individual vs. probabilistic face-patch definitions (Figures 4D and S5C; Table S4) using a bootstrap analysis as follows:

Bootstrap-generated Cohen’s d distributions of each face-patch pair were compared between the three contrasts outlined above. First, a distribution of staining intensities for each face patch was generated by calculating the mean intensity across depth bins for every section. Subsequently, based on this distribution, 10,000 new sets of sections were simulated to capture the variability in section staining for each face patch. In each of these 10,000 iterations, a new set of sections was created by randomly drawing intensity values with replacement from the original distribution derived from the actual data. Then, Cohen’s d was computed between the resampled section intensities of paired face patches for each iteration, yielding 10,000 Cohen’s d values for all paired face-patch comparisons, which simulate the variability of Cohen’s d within the sampled distributions for the same face-patch pair. For each contrast (e.g., CO vs. myelin), the bootstrap-generated Cohen’s d were calculated for the two features assessed, and these distributions were then statistically compared using the KS test.

##### Distance-based histological 2D mapping in posterior-anterior and dorsolateral-ventrolateral axes

We calculated mean staining intensities across cortical depth bins along a large segment of the STS that included ML, AL, and AF/AD face patches to visualize the spatial histo-architectonic organizations. Sagittal sections (right hemispheres) covered the posterior-to-anterior extent of the STS from the gyral banks partly into the fundus in both monkeys. Coronal sections (left hemispheres) covered the dorsal and ventral banks of the STS, with the posterior-to-anterior coverage varying between the monkeys (Figure S2). For each section, the STS was segmented into columnar bins running perpendicular to the cortical surface using Laynii’s LN_COLUMNAR_DIST,^22^ which calculates the distance along the middle layer within a defined ROI (i.e., STS) and then assigns all pixels in other layers the distance values of their nearest middle layer pixels. Staining intensities were averaged across depth bins to obtain a single intensity measure for each columnar bin. To generate a rectangular 2D histological map varying along the anterior-posterior and dorsolateral-ventrolateral axes, the lengths of STS segments were normalized across sections and then concatenated. Sagittal sections excluded the fundus and surrounding tissue of the STS. Outlines of face patches were overlaid on the 2D map.

To test if the staining intensity difference between the face patches were due to the broad histological gradient, we compared the staining intensities of face patches after subtracting the intensities of the neighboring STS regions outside the face patches (non-face patch). The staining intensities for both face patches and non-face patch regions were calculated by averaging the intensities of the columnar bins within and outside of the face patch ROIs at the corresponding positions across sections. For the sagittal map, the intensities were averaged at the same position along the posterior-anterior axis; for the coronal map, the intensities were averaged at the same position along the dorsolateral-ventrolateral axis (Figure S3 for the 1D plots of the two axes). We then subtracted the non-face patch region’s intensity from the face patch intensity for columnar bins at corresponding positions along these axes. We compared these resulting intensity differences between the face patches using unpaired, two-sample t-tests (Figure 4A).

## Supplementary Material

1

2

3

Supplemental information can be found online at https://doi.org/10.1016/j.celrep.2024.114732.

## Figures and Tables

**Table:** KEY RESOURCES TABLE

REAGENT or RESOURCE	SOURCE	IDENTIFIER

Antibodies		

Catalase	Sigma-Aldrich	Cat#9001–05-2; RRID:AB_991692
Cytochrome *c*	Sigma-Aldrich	Cat#9007–43-6; RRID:AB_1645293
3,3′-diaminobenzidine	Sigma-Aldrich	Cat#868272–85-9; RRID:AB_2335241

Chemicals, peptides, and recombinant proteins		

Silver nitrate	Sigma-Aldrich	Cat#7761–88-8
Ammonium nitrate	Thermo Scientific Chemicals	Cat#6484–52-2
Sodium hydroxide	J.T. Baker	Cat#1310–73-2
Sodium carbonate	J.T. Baker	Cat#497–19-8
Sodium thiosulfate	Sigma-Aldrich	Cat#7772–98-7
Tungstosilicic acid hydrate	Sigma-Aldrich	Cat#12027–43-9

Deposited data		

Histological section and face patch data	This study	https://osf.io/wjyb4/

Experimental models: organisms/strains		

Macaque monkey	Harvard Medical School	Macaca mulatta; RRID:NCBITaxon_9544

Software and algorithms		

MATLAB	MathWorks	http://www.mathworks.com; RRID:SCR_001622
FreeSurfer	FreeSurfer	https://surfer.nmr.mgh.harvard.edu/; RRID:SCR_001847
AFNI	Cox.^61^	https://afni.nimh.nih.gov; RRID:SCR_005927
SUMA	Saad and Reynolds.^62^	https://afni.nimh.nih.gov/Suma
JIP Analysis Toolkit	The General Hospital Corporation	https://www.nmr.mgh.harvard.edu/jbm/jip/; RRID:SCR_009588
LayNii	Huber et al.^22^	https://github.com/layerfMRI/LAYNII
Histo-architectonic depth profile	This study	https://osf.io/wjyb4/

## Highlights

- Integration of *in vivo* fMRI and postmortem histology in the same individuals
- IT face patches show different CO architecture, particularly in ML outer layers
- Precision mapping of histo-architecture is more accurate than probabilistic definitions
