# Supplementary material for: Inferotemporal face patches are histo-architectonically distinct
Source: Cell Rep. Author manuscript; Available in PMC 2025 Aug 29. (PMC12396101; doi:10.1016/j.celrep.2024.114732)
Supplement: 1 [file NIHMS2025379-supplement-1.pdf]

Cell Reports, Volume *43*

Supplemental information

Inferotemporal face patches

are histo-architectonically distinct

Hiroki Oishi, Vladimir K. Berezovskii, Margaret S. Livingstone, Kevin S. Weiner, and Michael J. Arcaro

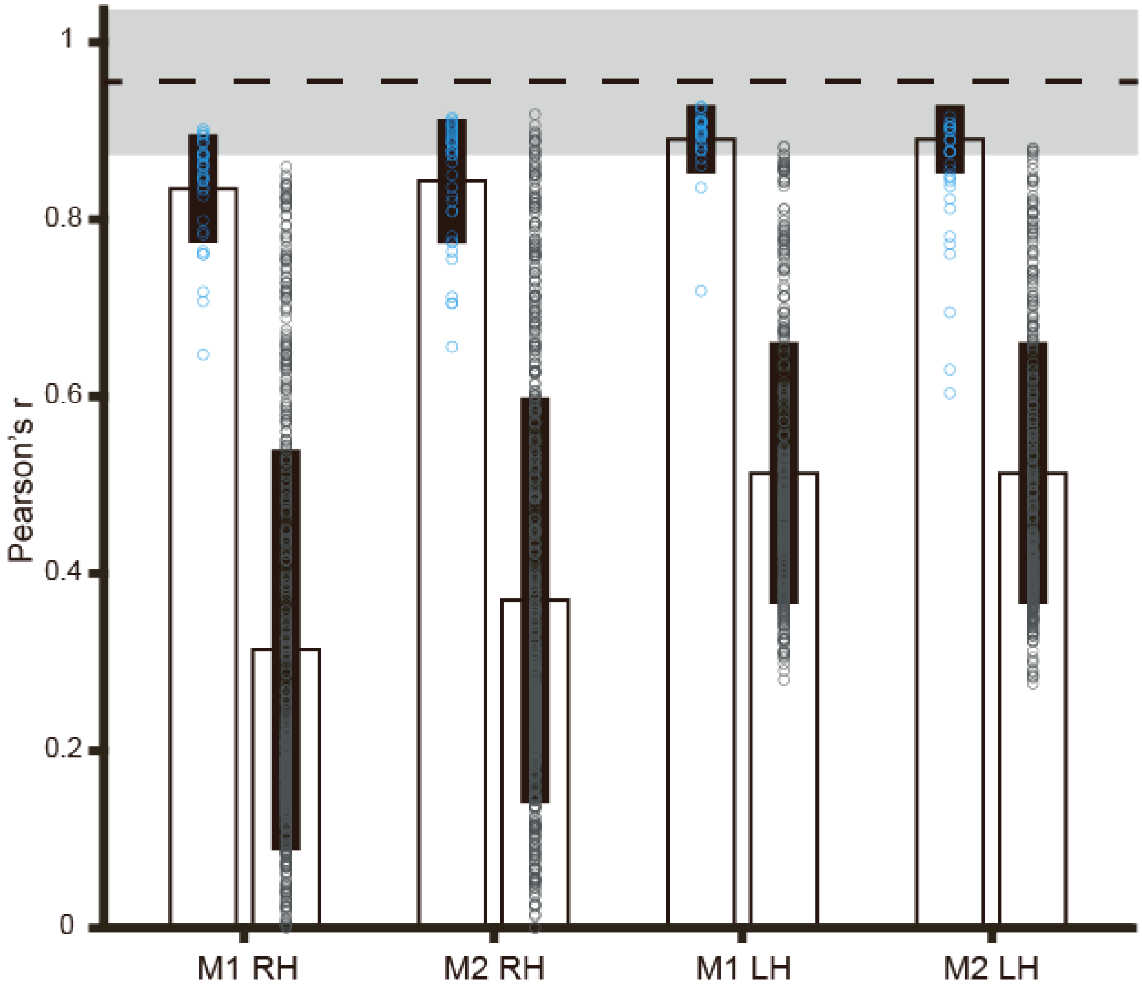

Figure S1. Accuracy of co-registration between MRI and histology in all hemispheres, Related to Figure 1.

Cyan and gray circles represent slice-by-slice correlations of the image intensities between corresponding and different slices of MRI and histology images, respectively. Bars represent the mean Pearson correlations (error bars, ±1 standard deviation). The correlations of corresponding slice pairs are significantly higher than those of different slice pairs (all ts > 12.263, all ps < 0.001), indicating high accuracy of co-registration between MRI and histology. The horizontal dashed black line (and shaded area) corresponds to the mean (and standard deviation) slice-by-slice correlation between two co-registered MRIs acquired from different sessions of M1, indicating the practical highest correlation between two MRI brain images.

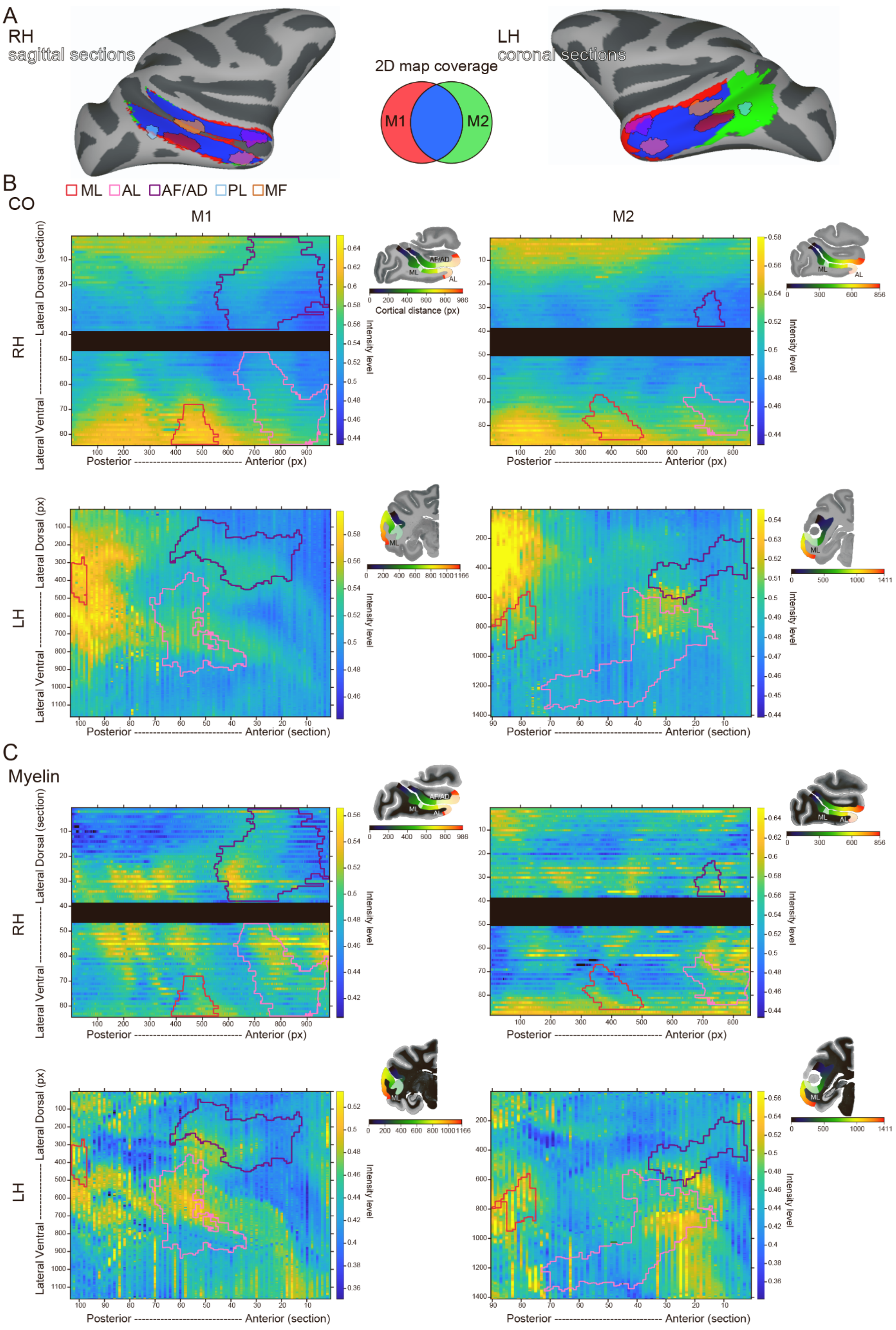

A
RH
sagittal sections
2D map coverage
M1
M2
LH
coronal sections
B
ML AL AF/AD PL MF
CO
M1
M2
RH
LH
Lateral Dorsal (section)
Lateral Ventral
Lateral Dorsal (px)
Posterior Anterior (px)
Posterior Anterior (section)
Intensity level
Cortical distance (px)
AF/AD
ML
AL
C
Myelin

Figure S2. CO and myelin intensity maps in and around the STS, Related to Figure 3.
(A) Cortical coverage of the 2D histological maps for all hemispheres (M1: red; M2: green; overlap: blue). Face patches are overlaid on the surface (ML: red; AL: pink; AF/AD: purple; PL: light blue; MF: orange). Note that PL and MF are excluded for the histological analyses because more than half their ROIs were outside the histology sections in at least half the hemispheres.
(B-C) Mean CO (B) and myelin (C) stain intensities averaged across depth bins in each column bin mapped for each section from posterior to anterior and from dorsolateral to ventrolateral for right hemispheres (sagittal sections; upper panels) and left hemispheres (coronal sections; lower panels). See Methods section, 'Distance-based histological 2D mapping in posterior-anterior and dorsolateral-ventrolateral', for the details of the map generation. The contours of the face patches are overlaid (ML: red; AL: pink; AF/AD: purple). Sagittal sections did not cover the fundus of the STS (black rectangle). The color gradient depicted in each upper-right representative section represents the pixel-wise cortical distance of the STS region.

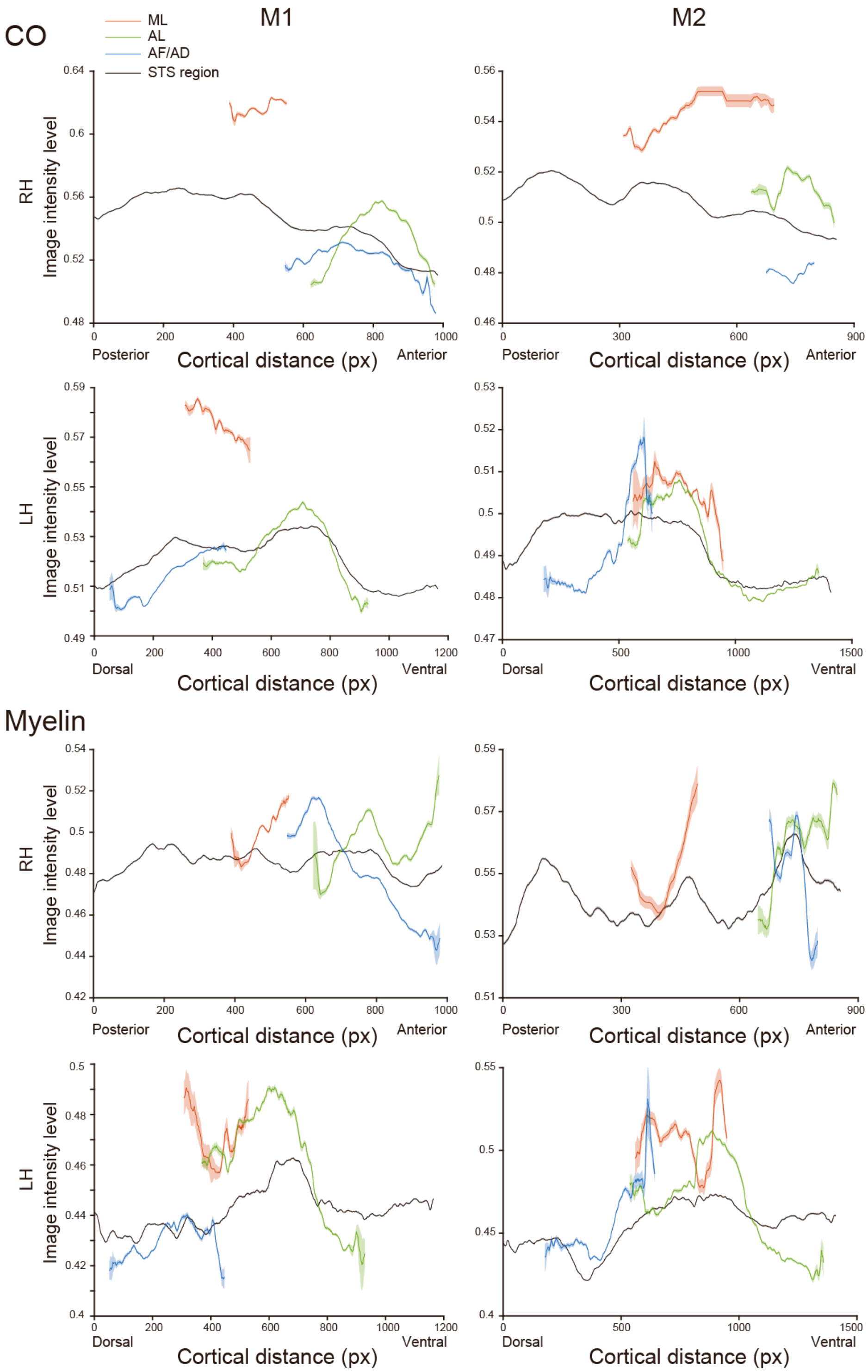

Figure S3. CO and myelin intensities of face patches and STS along posterior-anterior and dorsolateral-ventrolateral, Related to Figure 3. CO and myelin stain intensities, averaged across columnar bins at corresponding positions along sections of the STS (Figure S2B), are plotted (black lines). For the sagittal plots, intensities were averaged at the same position along the posterior-anterior axis; for the coronal map, the intensities were averaged at the same position along the dorsolateral-ventrolateral axis. Staining intensities for face patches are plotted (ML: red; AL: green; AF/AD: blue) separate from the surrounding STS (black). The shaded areas represent ±1 S.E.M. across sections.

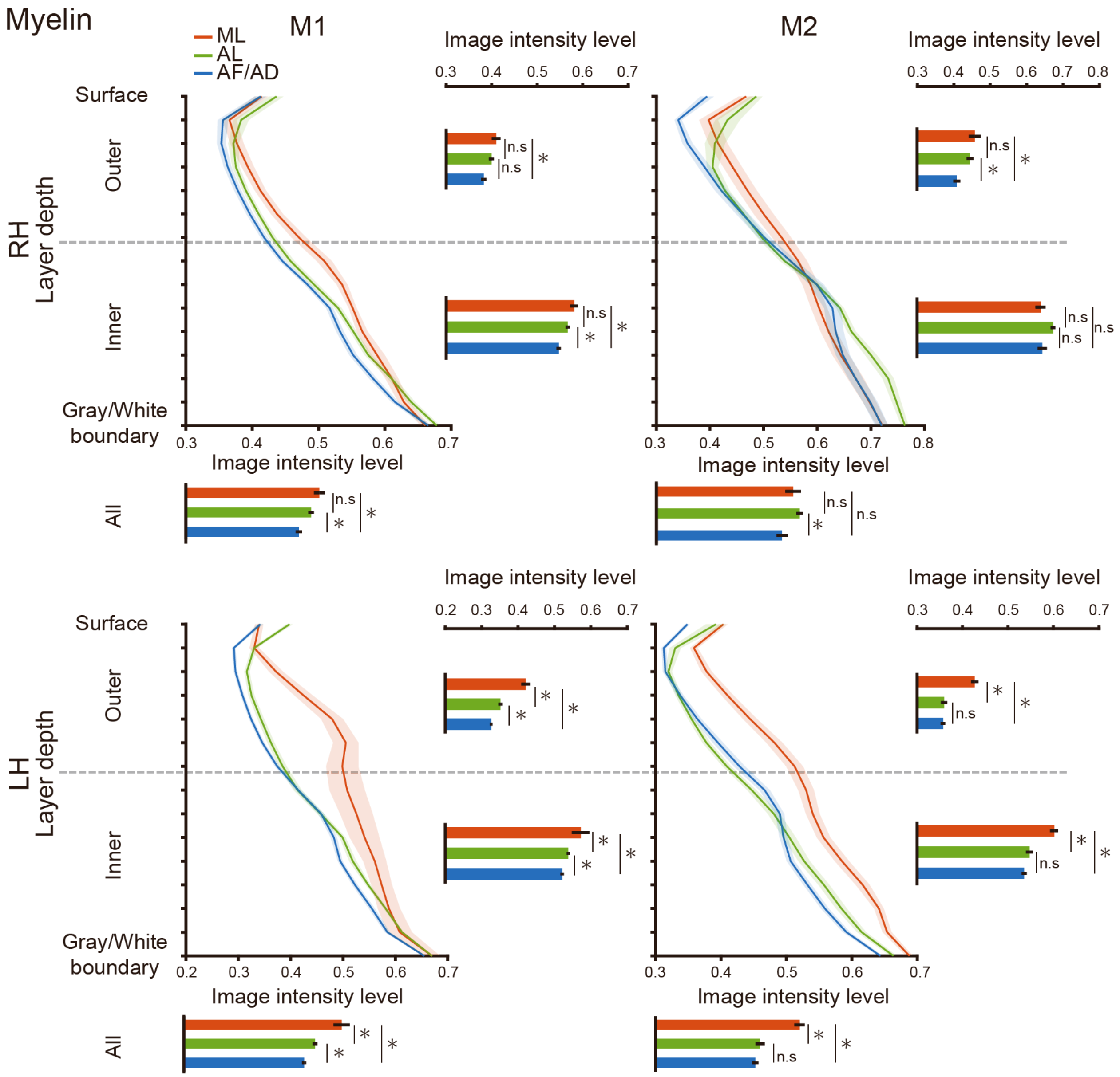

Figure S4. Myelin profiles of the face patches, Related to Figure 3.
Conventions consistent with Figure 3. The statistics of all comparisons are shown in Table S1.

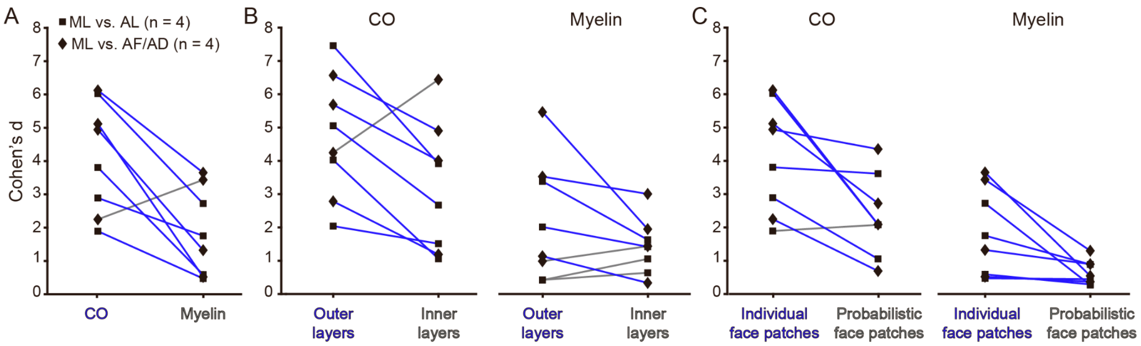

Figure S5. Cohen's d comparison of all hemispheres/monkeys shown in Fig. 4, Related to Figure 4.
The squares (ML vs. AL) and diamonds (ML vs. AF/AD) illustrate Cohen's d for individual hemispheres / monkeys. Symbols connected by blue lines indicate the greater architectonic effect for (A) CO- vs. myelo-architecture, (B) outer vs. inner layers, (C) individually vs. probabilistically defined face patches (all ps < 0.001, using KS test). Symbols connected by gray lines indicate the opposite significance (i.e., weaker effect for those comparisons). Conventions consistent with Figure 4. All statistics for each pairwise comparison for each figure are described in Tables S2-4.

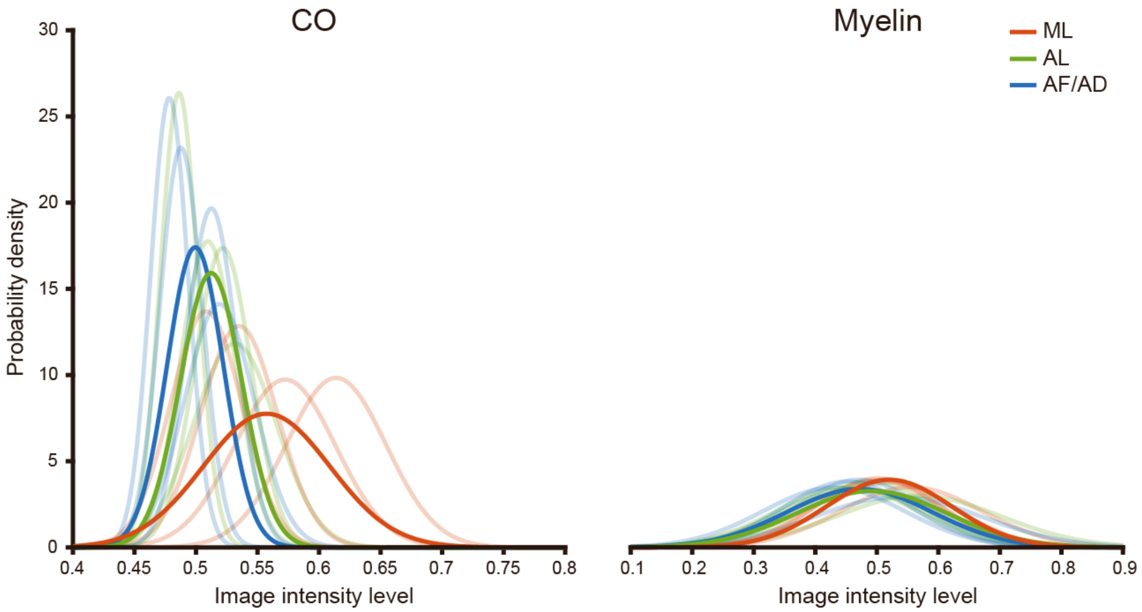

Figure S6. Group-level differences in CO architecture between ML and other face patches is greater than myeloarchitecture, Related to Figure 3.
Probabilistic distributions of (left) CO and (right) myelin intensities for ML, AL, AF/AD. Normal distributions were calculated for mean depth profiles of each face patch collapsed across monkeys and hemispheres shown in bold curves (ML, orange; AL, green, AF/AD, blue). Normal distributions were also calculated for all depth profiles of each face patch (n = 66 [15 × 11; depth bins × sections] for ML of M1's right hemisphere for example [Figue 2E]) in each monkey/hemisphere, shown in pale corresponding colors.

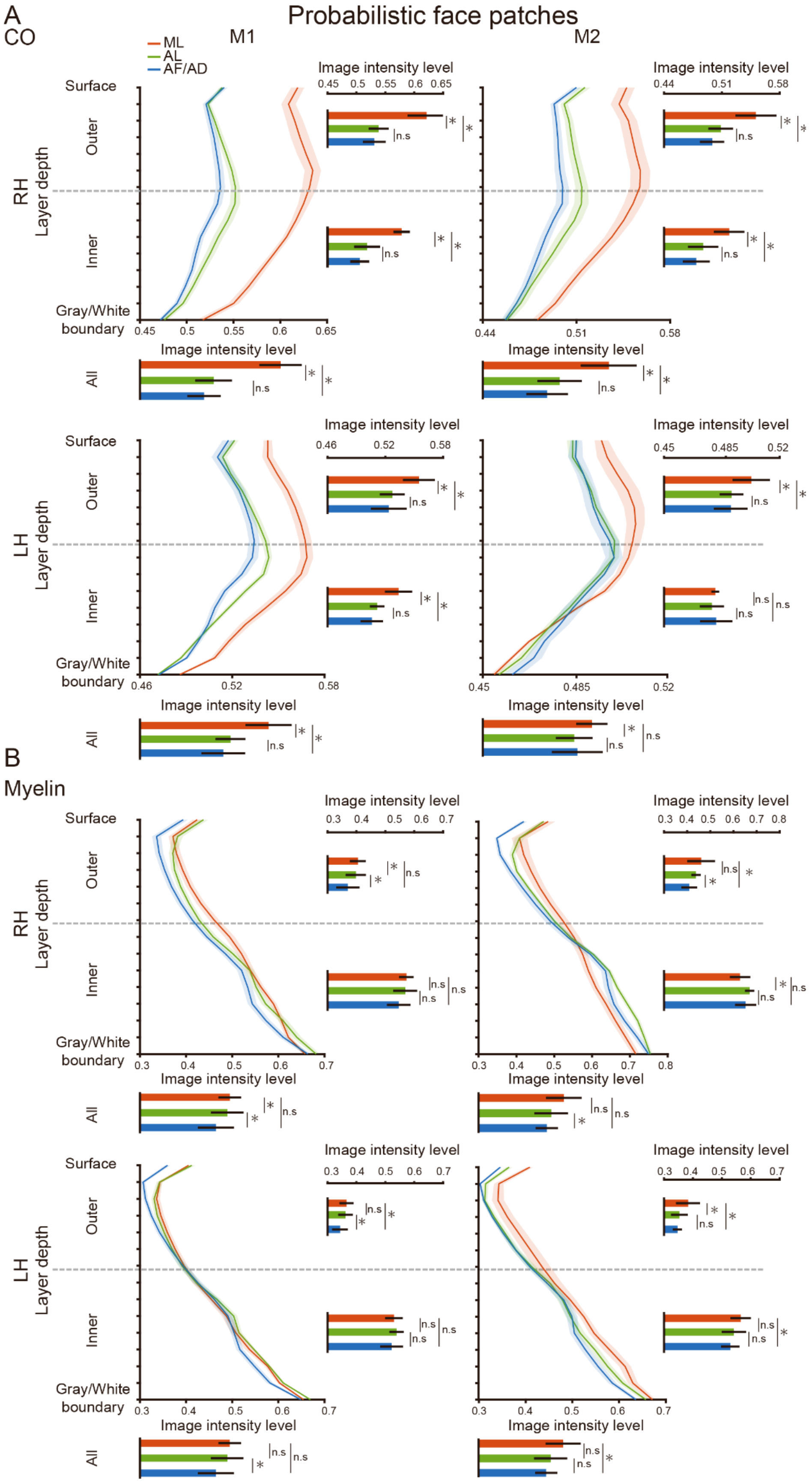

A
CO
Probabilistic face patches
M1
M2
ML
AL
AF/AD
Image intensity level
Surface
Outer
Inner
Gray/White boundary
RH
LH
Layer depth
All
n.s
*
B
Myelin

Figure S7. CO and Myelin profiles of the probabilistic face patches and their probabilistic distributions, Related to Figure 4.
(A) and (B) CO and myelin profiles are calculated in the probabilistically defined face patches. Conventions consistent with Figure 3. The statistics of all comparisons are shown in Table S1.

Face patch definition	Staining method	Monkey /hemisphere	Layer	ML vs. AL t (df)	ML vs. AL p	ML vs. AF/AD t (df)	ML vs. AF/AD p	AL vs. AF/AD t (df)	AL vs. AF/AD p
Individual	CO	M1 RH	Whole layer	10.165 (35)	<0.001*	13.127 (35)	<0.001*	1.890 (50)	0.096
			Outer layer	13.453 (35)	<0.001*	15.141 (35)	<0.001*	1.840 (50)	0.101
			Inner layer	7.097 (35)	<0.001*	10.602 (35)	<0.001*	1.876 (50)	0.097
		M2 RH	Whole layer	4.223 (21)	0.001*	11.801 (24)	<0.001*	8.412 (23)	<0.001*
			Outer layer	4.509 (21)	<0.001*	9.919 (24)	<0.001*	9.054 (23)	<0.001*
			Inner layer	3.452 (21)	0.006*	14.469 (24)	<0.001*	7.107 (23)	<0.001*
		M1 LH	Whole layer	10.131 (21)	<0.001*	10.655 (28)	<0.001*	3.197 (43)	0.006*
			Outer layer	12.693 (21)	<0.001*	11.574 (28)	<0.001*	2.304 (43)	0.044*
			Inner layer	6.309 (21)	<0.001*	8.359 (28)	<0.001*	4.221 (43)	<0.001*
		M2 LH	Whole layer	7.047 (37)	<0.001*	5.058 (24)	<0.001*	-0.759 (47)	0.504
			Outer layer	9.786 (37)	<0.001*	6.231 (24)	<0.001*	-1.939 (47)	0.088
			Inner layer	2.559 (37)	0.028*	2.626 (24)	0.028*	0.543 (47)	0.629
	Myelin	M1 RH	Whole layer	1.501 (33)	0.185	3.386 (33)	0.0045*	2.922 (48)	0.012*
			Outer layer	0.984 (33)	0.399	2.545 (33)	0.029*	2.152 (48)	0.058
			Inner layer	1.602 (33)	0.160	3.679 (33)	0.002*	2.949 (48)	0.011*
		M2 RH	Whole layer	-0.747 (21)	0.509	1.190 (25)	0.302	2.437 (24)	0.039*
			Outer layer	0.682 (21)	0.544	2.779 (25)	0.021*	2.652 (24)	0.027*
			Inner layer	-2.172 (21)	0.065	-0.239 (25)	0.825	1.840 (24)	0.108
		M1 LH	Whole layer	4.021 (21)	0.002*	5.690 (28)	<0.001*	3.535 (45)	0.003*
			Outer layer	5.023 (21)	<0.001*	8.151 (28)	<0.001*	4.200 (45)	<0.001*
			Inner layer	2.498 (21)	0.036*	3.097 (28)	0.001*	2.446 (45)	0.032*
		M2 LH	Whole layer	4.207 (37)	<0.001*	7.473 (23)	<0.001*	0.736 (46)	0.509
			Outer layer	4.759 (37)	<0.001*	7.588 (23)	<0.001*	0.245 (46)	0.825
			Inner layer	3.371 (37)	0.004*	6.538 (23)	<0.001*	1.040 (46)	0.368
Probabilistic	CO	M1 RH	Whole layer	10.168 (35)	<0.001*	12.276 (35)	<0.001*	1.871 (44)	0.098
			Outer layer	10.091 (35)	<0.001*	10.509 (35)	<0.001*	1.350 (44)	0.228
			Inner layer	8.975 (35)	<0.001*	13.783 (35)	<0.001*	2.203 (44)	0.053
		M2 RH	Whole layer	5.356 (28)	<0.001*	7.397 (32)	<0.001*	1.750 (34)	0.122
			Outer layer	5.780 (28)	<0.001*	7.820 (32)	<0.001*	2.109 (34)	0.065
			Inner layer	4.683 (28)	<0.001*	6.652 (32)	<0.001*	1.461 (34)	0.193
		M1 LH	Whole layer	6.603 (42)	<0.001*	6.816 (44)	<0.001*	1.353 (48)	0.228
			Outer layer	6.222 (42)	<0.001*	5.961 (44)	<0.001*	0.817 (48)	0.478
			Inner layer	6.692 (42)	<0.001*	7.332 (44)	<0.001*	1.981 (48)	0.081
		M2 LH	Whole layer	2.573 (27)	0.029*	1.584 (21)	0.171	-0.399 (30)	0.718
			Outer layer	3.488 (27)	0.004*	2.767 (21)	0.023*	0.150 (30)	0.881
			Inner layer	0.854 (27)	0.464	-0.173 (21)	0.870	-0.861 (30)	0.463
	Myelin	M1 RH	Whole layer	0.490 (34)	0.660	2.491 (33)	0.032*	2.232 (43)	0.050*
			Outer layer	0.618 (34)	0.581	2.732 (33)	0.021*	2.449 (43)	0.032*
			Inner layer	0.317 (34)	0.775	2.109 (33)	0.065	1.836 (43)	0.102
		M2 RH	Whole layer	-0.948 (30)	0.417	0.776 (32)	0.503	2.320 (34)	0.044*
			Outer layer	1.474 (30)	0.193	3.174 (32)	0.008*	3.069 (34)	0.010*
			Inner layer	-3.532 (30)	0.003*	-1.552 (32)	0.172	1.467 (34)	0.193
		M1 LH	Whole layer	-0.414 (42)	0.711	1.713 (43)	0.128	2.349 (49)	0.039*
			Outer layer	0.488 (42)	0.660	2.865 (43)	0.014*	2.545 (49)	0.027*
			Inner layer	-1.070 (42)	0.355	0.760 (43)	0.504	1.854 (49)	0.099
		M2 LH	Whole layer	1.914 (34)	0.097	2.840 (33)	0.020*	0.848 (43)	0.464
			Outer layer	2.322 (34)	0.045*	2.923 (33)	0.017*	0.737 (43)	0.509
			Inner layer	1.525 (34)	0.181	2.565 (33)	0.032*	0.881 (43)	0.455

Table S1. All comparisons of CO and myelin profiles between face patches, Related to Figure 3. Comparisons between face patches were performed using unpaired, two-sample t-test collapsed across layers (whole layer), in outer layers, and in inner layers, for individually defined face patches as well as probabilistically defined face patches. FDR correction was applied to account for multiple comparisons (* < 0.05).

Monkey /hemisphere	ML vs. AL			ML vs. AF/AD			AL vs. AF/AD		
	Cohen's d Δ	KS distance	p	Cohen's d Δ	KS distance	p	Cohen's d Δ	KS distance	p
M1 RH	3.216	1.000	<0.001*	3.618	1.000	<0.001*	-0.323	0.415	<0.001*
M2 RH	1.421	0.8805	<0.001*	4.598	1.000	<0.001*	2.619	0.999	<0.001*
M1 LH	3.301	0.893	<0.001*	2.471	0.820	<0.001*	-0.089	0.135	<0.001*
M2 LH	1.137	0.799	<0.001*	-1.187	0.684	<0.001*	0.045	0.071	<0.001*

Table S2. Effect size (Cohen's d) comparisons between CO and myelin, Related to Figure 4. Bootstrap-generated Cohen's ds were compared between CO and myelin for each face-patch pair using KS test. FDR correction was applied to account for multiple comparisons (* < 0.05). Cohen's d Δ represents the difference between the mean Cohen's d of CO and myelin, with positive values indicating a higher Cohen's d of CO than myelin.

Staining method	Monkey /hemisphere	ML vs. AL			ML vs. AF/AD			AL vs. AF/AD		
		Cohen's d Δ	KS distance	p	Cohen's d Δ	KS distance	p	Cohen's d Δ	KS distance	p
CO	M1 RH	2.382	0.975	<0.001*	1.681	0.783	<0.001*	0.007	0.018	0.069
	M2 RH	0.527	0.366	<0.001*	-2.194	0.659	<0.001*	0.798	0.569	<0.001*
	M1 LH	3.542	0.828	<0.001*	1.664	0.650	<0.001*	-0.602	0.748	<0.001*
	M2 LH	2.965	0.990	<0.001*	1.594	0.882	<0.001*	0.316	0.438	<0.001*
Myelin	M1 RH	-0.214	0.270	<0.001*	-0.451	0.467	<0.001*	-0.226	0.295	<0.001*
	M2 RH	-0.635	0.541	<0.001*	0.801	0.784	<0.001*	0.333	0.394	<0.001*
	M1 LH	1.754	0.714	<0.001*	3.516	0.932	<0.001*	0.559	0.610	<0.001*
	M2 LH	0.594	0.491	<0.001*	0.522	0.271	<0.001*	-0.128	0.278	<0.001*

Table S3. Cohen's d comparisons between outer and inner layers, Related to Figure 4. Bootstrap-generated Cohen's ds of upper 7 layer bins and lower 8 bins were compared in each face-patch pair in CO and myelin using KS test. FDR correction was applied to account for multiple comparisons (* < 0.05). Cohen's d Δ represents the difference between the mean Cohen's d of outer and inner layers, with positive values indicating a higher Cohen's d of outer than inner layers.

Staining method	Monkey /hemisphere	ML vs. AL			ML vs. AF/AD			AL vs. AF/AD		
		Cohen's d Δ	KS distance	p	Cohen's d Δ	KS distance	p	Cohen's d Δ	KS distance	p
CO	M1 RH	0.192	0.226	<0.001*	0.589	0.341	<0.001*	-0.033	0.052	<0.001*
	M2 RH	-0.184	0.211	<0.001*	2.395	0.911	<0.001*	3.020	1.000	<0.001*
	M1 LH	3.939	0.998	<0.001*	4.033	1.000	<0.001*	0.583	0.755	<0.001*
	M2 LH	1.838	0.977	<0.001*	1.557	0.964	<0.001*	-0.025	0.041	<0.001*
Myelin	M1 RH	0.302	0.421	<0.001*	0.426	0.501	<0.001*	0.177	0.227	<0.001*
	M2 RH	0.028	0.029	<0.001*	0.151	0.220	<0.001*	0.203	0.280	<0.001*
	M1 LH	2.454	0.990	<0.001*	3.107	0.999	<0.001*	0.400	0.502	<0.001*
	M2 LH	0.865	0.689	<0.001*	2.136	0.930	<0.001*	-0.090	0.168	<0.001*

Table S4. Cohen's d comparison between the approaches using individually defined face patches vs. probabilistically defined face patches, Related to Figure 4.

Bootstrap-generated Cohen's ds when using individually and probabilistically defined face patches were compared for each face-patch pair in both CO and myelin using KS test. FDR correction was applied to account for multiple comparisons (* < 0.05). Cohen's d Δ represents the difference between the mean Cohen's d when using individually and probabilistically defined face patches, with positive values indicating a higher Cohen's d when using individually defined face patches.

M1_RH_MRI_histology_LM.mp4

Video S1. Sagittal scroll view of the aligned MRI and histological volume of sagittal sections, Related to Figure 1.
M1's MR anatomical volume (Left), which was resampled and aligned to its histological volume of sagittal sections (Right). This video scrolls the images of the aligned MRI and histological volume from lateral to medial in a resolution of histological volume. Staining intensities of myelin sections are inverted for the consistent visualization with CO sections.

M1_RH_MRI_histology_PA.mp4

Video S2. Coronal scroll view of the aligned MRI and histological volume of sagittal sections, Related to Figure 1.
This video scrolls the same anatomical MRI and histological data of Video S1 from posterior to anterior. This video demonstrates the sufficient alignment between MRI and histological data not only in the sagittal axis but also in the coronal axis.
